# Cytotoxicity and Initial Biocompatibility of Endodontic Biomaterials (MTA and Biodentine*™*) Used as Root-End Filling Materials

**DOI:** 10.1155/2016/7926961

**Published:** 2016-08-09

**Authors:** Diana María Escobar-García, Eva Aguirre-López, Verónica Méndez-González, Amaury Pozos-Guillén

**Affiliations:** ^1^Basic Sciences Laboratory, Faculty of Dentistry, San Luis Potosi University, 78290 San Luis Potosí, SLP, Mexico; ^2^Endodontics Posgraduated Program, Faculty of Dentistry, San Luis Potosi University, 78290 San Luis Potosí, SLP, Mexico

## Abstract

*Objective*. The aim of this study was to evaluate the cytotoxicity and cellular adhesion of Mineral Trioxide Aggregate (MTA) and Biodentine (BD) on periodontal ligament fibroblasts (PDL).* Methods*. PDL cells were obtained from nonerupted third molars and cultured; MTS cellular profusion test was carried out in two groups: MTA and BD, with respective controls at different time periods. Also, the LIVE/DEAD assay was performed at 24 h. For evaluation of cellular adhesion, immunocytochemistry was conducted to discern the expression of Integrin *β*1 and Vinculin at 12 h and 24 h. Statistical analysis was performed by the Kruskal-Wallis and Mann-Whitney* U* tests.* Results*. MTA and BD exhibited living cells up to 7 days. More expressions of Integrin *β*1 and Vinculin were demonstrated in the control group, followed by BD and MTA, which also showed cellular loss and morphological changes. There was a significant difference in the experimental groups cultured for 5 and 7 days compared with the control, but there was no significant statistical difference between both cements.* Conclusions*. Neither material was cytotoxic during the time evaluated. There was an increase of cell adhesion through the expression of focal contacts observed in the case of BD, followed by MTA, but not significantly.

## 1. Introduction

The objective of a cement placed at the root end is to provide apical sealing that prevents bacterial flow, bacterial products, and irritants from the root canal toward periapical tissues and from the tissue to root canal [1, 2]; this material must be biocompatible with periradicular tissues [2–4].

Currently, the most frequently used root-end filling cement is Mineral Trioxide Aggregate (MTA), which is composed of dicalcium and tricalcium silicate, tricalcium aluminate, calcium sulfate dehydrate, and bismuth oxide. It is produced from fine hydrophobic particles that harden in the presence of humidity [5]. MTA is characterized by its biocompatibility, radiopacity, and resistance, in comparison with other root-end filling materials, such as amalgam, Super-EBA, and IRM [6–8]. It requires an estimated time of 5 minutes (min) of work and a setting time that ranges from 2 hours (h) and 45 min to 4 h. Its cementogenic activity occurs due to its release of calcium ions, which interact with phosphate groups in the fluids surrounding the tissues to form hydroxyapatite on the surface of MTA [4]. It has disadvantages, such as long time to seal, low resistance to compression, and low flow capacity [8].

Biodentine (BD) is a bioactive material that can be employed for these different purposes, in addition to its offering improvement over the characteristics of MTA in terms of manipulation, compatibility, and hardening [4, 9, 10]. BD has been proposed with a dentine substitute [11]. Its compounds in terms of a powder component of the material consist of tricalcium silicate, a dicalcium silicate compound better than Portland and MTA cement, calcium carbonate and oxide filler, and iron oxide shade and zirconium oxide (as radiopacifier); the liquid contains calcium chloride as an accelerator and a hydrosoluble polymer (as a water-reducing agent) [12, 13]. This product controls the purity of the calcium silicate by removing the aluminum and other pollutants; it also increases physicochemical properties such as rapid hardening. Calcium silicate purity is controlled by removing aluminum and other impurities, thus increasing physicochemical properties such as rapid curing, high mechanical strength, and an initial setting time of 6 min and final setting time of 10–12 min; this is an improvement compared with the high-density glass ionomer and MTA [12].

When a novel material emerges, it must be evaluated by the conduction of various tests* in vitro*. The first tests to be performed* in vitro* are cytotoxicity tests, which estimate the possible alteration in basic cellular functions leading to damage that can be evaluated by detecting distinct cellular level-behavior metabolism, structure, and properties essential for cell survival, such as proliferation and cell function [11]. A second test is that of biocompatibility, in which the ability of a restorative material is evaluated to induce an appropriate and advantageous response to the host's response during clinical use [14]. In this study, an immunocytochemical test that allowed detection of specific antigens in fibroblasts was performed. In this test, antigen-antibody binding comprises a highly specific process; thus, this method is reliable for protein localization to focal adhesion complexes [13]. The aim of this study was to evaluate the cytotoxicity and the cellular adhesion of MTA and BD on periodontal ligament fibroblasts (PDL).

## 2. Materials and Methods

Third molars were used, donated voluntarily by patients attending at the Clinic of Oral and Maxillofacial Surgery. The patients or guardians of these patients previously signed informed consent, agreeing to participate in the study, which was approved by the Institutional Ethics Committee. The teeth obtained were disinfected with 2% chlorhexidine gluconate (IndiSpense® Refill; Ultradent Products, Inc., USA).

### 2.1. First Culture of Periodontal Ligament Fibroblast

Periodontal tissue was removed by root scraping. The extracted tissue was placed into a 2 mL microtube containing transport medium (Phosphate-buffered solution [PBS]) with 3% of antibiotics (1,000 U/mL Penicillin, 1 mg/mL Streptomycin, and Amphotericin B, 2.5 mg/*μ*L). The tissue was labeled and stored at 4°C for a period of 6–12 h for subsequent processing in the laboratory.

### 2.2. Cell Culture

The samples were washed with sterile PBS and incubated for 4–6 h with 2 mg/mL collagenase 1 (Sigma-Aldrich BioSciences, St. Louis, MO, USA); after this time, the periodontal tissue was dissected into pieces of approximately 1–3 mm per explant and plated on 25 mL cell box cultures with 3 mL of culture medium (Dulbecco's Modified Eagle's Medium [DMEM]-D6046; Sigma-Aldrich BioSciences, St. Louis, MO, USA), supplemented with 10% fetal bovine serum (FBS) and 1% antibiotic, incubated at 37°C in an atmosphere of 95% humidity and 5% CO_2_, with a change of medium every third day. Cells were employed once they reached 80% confluence. When >80% confluence was observed, subcultures were performed. Monolayers of the cells adhered to the culture dishes were detached with the aid of Trypsin EDTA 0.25% 1x solution (Gibco, Life Technologies, USA) to assess cell count and viability (50,000 cells for each experiment).

### 2.3. Preparation of Culture Medium with the Different Biomaterials: MTA and BD

The two biomaterials (MTA and BD) were prepared according to the manufacturer's instructions. Once the sealants were prepared, they were mixed with supplemented medium to a concentration of 2.5 mg/mL and agitated overnight; the medium obtained was spun at 13,000 rpm for 5 min, the supernatant was recovered, the medium was spun, again, and the supernatant obtained at the second spin was filtered with 0.22 membrane and stored at 4°C for its later use, and this contained the products released from the sealants which allow evaluation of cytotoxicity and cell adhesion.

### 2.4. Cell Proliferation Assay (MTS)

CellTiter 96® Aqueous Nonradioactive Cell Proliferation is a colorimetric assay used to measure the number of viable cells in proliferation or chemosensitive cells. Once the cell cultures are found at 80–90% confluence, they were detached with trypsin; 50,000 cells were plated in 100 *μ*L of culture medium onto 96-well microplates and these were incubated during a 4 h period at 37°C, 5% CO_2_, and 95% humidity. For this test, two groups of fibroblasts treated with different sealants were evaluated: (i) MTA-Angelus (Industria de Productos Odontológicos, Londrina, PR, Brazil); (ii) BD (Septodont, St. Maur-des-Fossés, France), and a control group. They were evaluated by 12, 24, and 48 h, and at 5 and 7 days. Initially, culture medium was added without cement, to allow adaptation of the cells to the well for 24 h. After that time, the culture medium was removed and replaced with 100 *μ*L of culture medium with the cements evaluated (MTA and BD, resp.) and medium without cement for the control group. Upon expiration of the corresponding evaluation periods, a sufficient amount of working solution was prepared for each 2-mL rate of MTS solution, 100 *μ*L of PMS solution immediately prior to use was prepared (CellTiter 96 Aqueous Nonradioactive Cell Proliferation Assay, Promega Corporation), and 20 *μ*L was added to each well directly on cells. Then, this was incubated for a 4 h period at 37°C, 5% CO_2_, and 95% humidity; during this period, the metabolically active cells bioreduced MTS salt to formazan which is soluble in the culture medium; this reaction takes place in the mitochondria through the dehydrogenase enzymes and the assay was subsequently read on a microplate reader (Thermo Scientific FC Multiskan®, Vantaa, Finland) at 490 nm. The absorbance is directly proportional to the number of living cells in culture. All tests were compared with control cells untreated with sealants, and all dilutions were tested in triplicate.

### 2.5. LIVE/DEAD Test

Two- or 3-day cell cultures were performed (the time at which they reached appropriate fibroblast density) in 24-well culture boxes, on top of which circular coverslips (13 mm) have been previously placed. The cell culture was incubated with different root-end filling materials (MTA and BD) for 24 h at 37°C with 5% CO_2_ and 95% humidity; afterward, the cells were washed with PBS to remove the esterase present in the FBS utilized to enrich the treated culture medium in which the cells were grown. Live cells are distinguished by the presence of intracellular esterase activity with the addition of calcein, this cells present a green fluorescent. EthD-1 enters cells with damaged membranes and binds to nucleic acids thereby producing a red fluorescent in dead cells. The LIVE/DEAD working solution was prepared at concentrations of 2 *μ*M calcein and 4 *μ*M EthD-1 in PBS, according to the manufacturer's directions (Life Technologies LIVE/DEAD Viability/Cytotoxicity kit; Life Technologies, USA). Of this solution, 100 *μ*L was added directly to the cell culture, which was incubated for 45 min at room temperature. At the end of this time, the cells were washed with PBS and the coverslip-stained cells were removed and observed by confocal laser scanning microscope (CLSM) (DMI4000B; Leica Microsystems, Wetzlar, Germany). Images were processed and analyzed using the LASAF® software (Leica, Germany). Adherent live cells were identified and relative fluorescence was calculated based on 3 images at 40x acquired randomly. Two root-end filling materials were compared with a negative and positive control and tests were performed in triplicate.

### 2.6. Immunocytochemical Assay

The expression of Integrin *β*1 and Vinculin was evaluated in two groups as follows: (a) fibroblasts treated with MTA, (b) fibroblasts treated with BD, and control groups at 12 and 24 h; 8 × 10^−3^ cells per sample were plated onto the circular slide on which 24-well boxes were placed, culture medium was immediately added without cement in order to allow adhesion of the cells to the slide, and these were incubated for a period of 24 h at 37°C, 5% CO_2_, and 95% humidity. After this period, the culture medium was replaced with culture medium prepared with the cement and they were cultured for 12 and 24 hours, after each incubation period, the samples were fixed with 10% neutral Formalin, and the process was blocked with PBS with 1% albumin and 0.025% Tween prior to contact with the first antibody (Integrin *β*1 mouse monoclonal IgG 2a and Vinculin Monoclonal Mouse IgG1) in 1 : 100 dilutions; this incubation was for an overnight period. The following day, samples were washed with PBS and contacted with the second antibody, normal mouse IgG Alexa Fluor 488, in 1 : 500 dilutions; this process was carried out under conditions of darkness and incubated for 3 h at room temperature. Finally, the samples were washed with PBS and observed under CLSM and inhibition percent of Integrin *β*1 and Vinculin was calculated with respect to control.

### 2.7. Statistical Analysis

For comparing differences between experimental materials and controls at different periods of evaluation time, a Kruskal-Wallis test was used. To identify possible significant differences between the results obtained from the study groups, a nonparametric Mann-Whitney *U* test was used, with a significance level set at 0.05 (*p* < 0.05) in a two-tailed test (SigmaPlot Ver. 11; Systat Software, Inc., San Jose, CA, USA).

## 3. Results

In the cell proliferation assay, at 12 and 24 h, the majority of cell proliferation was found in the control group, followed by that in MTA-treated fibroblasts, and fibroblasts cultured with BD-supplemented medium exhibited a slight decrease in proliferation. At 48 h, the majority of cell proliferation was observed in fibroblasts cultured with MTA-supplemented medium; control fibroblasts and BD-treated fibroblasts demonstrated very similar behavior; however, these results were not statistically significant (*p* > 0.05).

**Figure 1:**
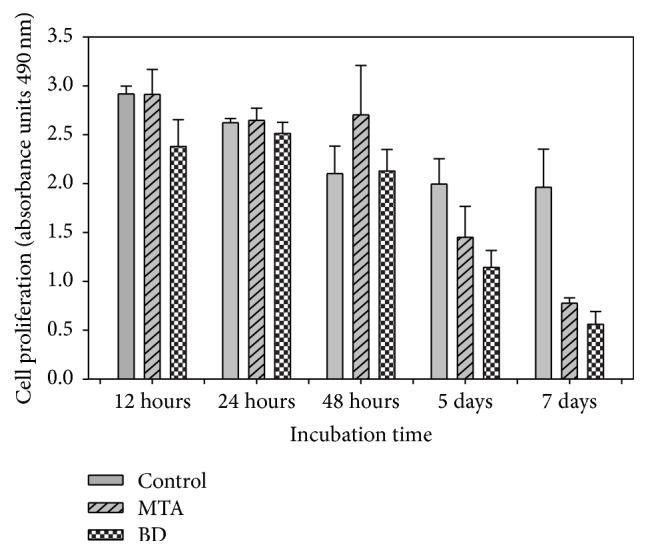
Fibroblast cell proliferation of human periodontal ligament in contact with Mineral Trioxide Aggregate (MTA) and Biodentine (BD) cements at different periods of time.

During the remaining time periods, evaluation showed a decrease in the percentage of cell viability; however, all groups presented viable cells at 7 days (MTA 39.51% and BD 28.58%). No statistically significant difference was found. There was a statistically significant difference in the experimental groups cultured for 5 (*p* = 0.018) and 7 (*p* < 0.001) days compared with the control, but there was no statistically significant difference between the two cements (Figure 1).

**Figure 2:**
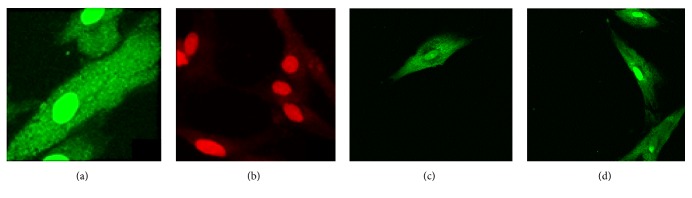
LIVE/DEAD® assay: (a) control without cement at 24 h; (b) control with H_2_O_2_ at 24 h; (c) fibroblast in contact with Mineral Trioxide Aggregate- (MTA-) enriched medium for 24 h, and (d) fibroblast in contact with Biodentine- (BD-) enriched medium for 24 h.

The LIFE/DEAD test was evaluated after a 24 h incubation period. MTA-treated cell cultures exhibited fewer cells in cultures compared with BD-treated cells (*p* < 0.05) (Table 1). This result was repeated in cell cultures maintained under treatment for 24 h with different biomaterials (Figure 2).

**Figure 3:**
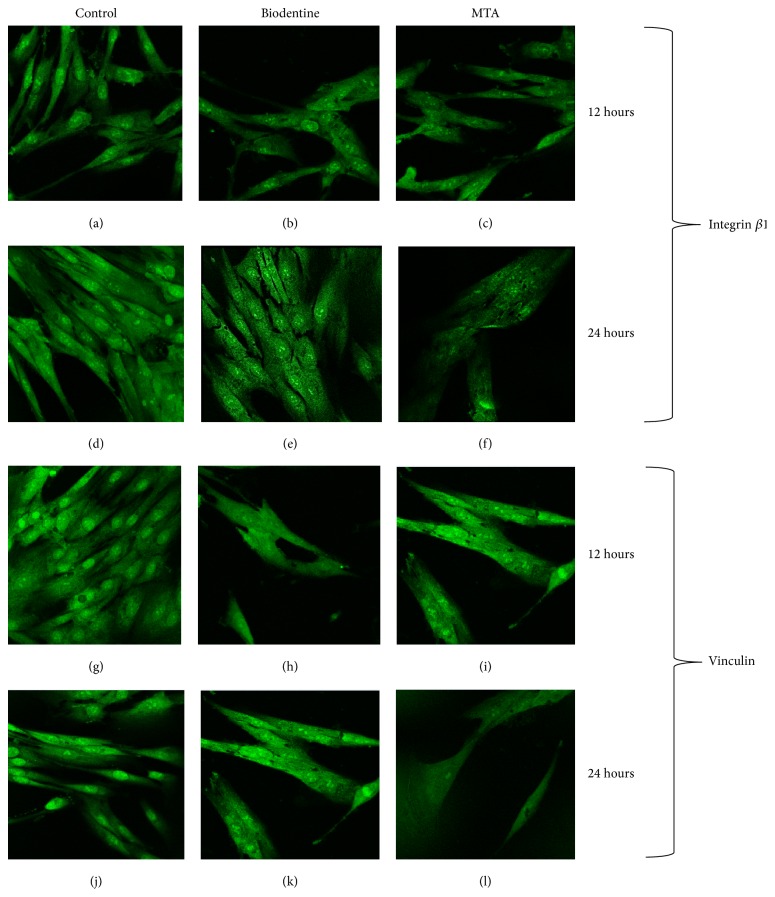
Confocal Laser MicroScope (CLMS) to detect Vinculin and Integrin *β*1 in fibroblast periodontal ligament following treatment with medium enriched with sealants. Integrin *β*1: (a) untreated fibroblasts cultured for 12 h; (b) Biodentine- (BD-) treated fibroblasts cultured for 12 h; (c) Mineral Trioxide Aggregates- (MTA-) treated fibroblasts for 12 h, (d) untreated fibroblasts cultured for 24 h; (e) cultured fibroblasts treated with BD for 24 h; (f) fibroblast cultured with MTA-enriched culture medium for 24 h. Vinculin: (g) untreated fibroblasts cultured for 12 h; (h) BD-treated fibroblasts cultured for 12 h; (i) fibroblasts treated with MTA for 12 h; (j) untreated fibroblasts cultured for 24 h; (k) treated fibroblasts grown by BD for 24 h; (l) fibroblasts cultured with MTA-enriched medium for 24 h.

The immunohistochemistry test was performed in cell cultures with two incubation periods (12 h and 24 h) with different culture media. Integrin *β*1 expression at 12 h was similar between the experimental groups and the control group, while, at 24 h, this expression was reduced, mainly in the MTA-treated group followed by group treated with BD (Figure 3). Similarly, exposure of fibroblasts to medium enriched with MTA and BD for 24 h caused morphological changes in cells and fewer projections and focal contacts (Figure 3). This result was repeated in tests in which Vinculin expression was evaluated (Figure 3), with fewer cellular adhesions to the substrate; these results were not statistically significant (*p* > 0.05) (Table 2).

## 4. Discussion

In this study the cytotoxicity of MTA and BD was evaluated in terms of cell viability using MTS, in which the result obtained was that viable cells remained in all evaluated periods and no statistically significant difference was found between both materials, showing greater similarity at 24 h and a higher percentage of cell proliferation at 48 h; the gradual increase in cell viability can be attributed to the constituents of the materials [15], because bioactive materials interact with the host tissue in a controlled way, which goes beyond the characteristic of biocompatibility [16]. Proliferation decreased at day 5, which continued until day 7. This decrease in cell number may be due to the stimulation occurring in development at 48 h because of the presence of cement; there was an increase in fibroblast proliferation without observing altered metabolism with either cement; on introducing greater confluence, the fibroblasts died during the competitive process, which gave rise to a decrease in the amount of nutrients necessary for their survival.

Results of the study are in agreement with those of Khedmat et al. [17], in which BD and MTA exhibited similar behavior to that of the other materials tested. Both MTA and BD demonstrated being non-cytotoxic and did not present a significant difference with regard to viability at 24 and 48 h; at 48 h, cells grown in the presence of MTA and BD possessed a higher percentage of viability than the groups treated with other cements, but there were no significant differences between BD and MTA (*p* < 0.05) [17–19].

Meanwhile, the remaining assays that measured the cytotoxicity of MTA and BD at three times (1, 3, and 5 days) employing the MTT assay found that both cements demonstrated viable cells; the highest percentage of viability was exhibited at 48 h and was decreasing at 72 h, consistent with the results obtained in this study [20–23].

In a next step of the cytotoxicity analysis and after assessing cell proliferation, the toxicity was observed of both cements based on membrane-damage fibroblasts that may cause and consequently generate cell death. This evaluation was carried out by the LIFE/DEAD test. Both treatment groups were treated for a period of 24 h with MTA and BD maintaining their membrane integrates, allowing the polyanionic calcein to be retained within the cells, generating an intense green color. Thus, MTA and BD were shown to be cytotoxic. These results confirm the data obtained with the cell proliferation test, in which it was observed that both materials are not cytotoxic.

In this study, cell adhesion was evaluated through the formation of focal contacts, in order to determine initial biocompatibility, with periodontal ligament fibroblasts versus both cements. This parameter was evaluated by immunocytochemistry because it is a specific test for the detection of Integrin *β*1 and Vinculin. Vinculin is an adhesion molecule and is localized on the surface of the cell membrane, which allows it, among other functions, to serve as an “anchor” to initiate and maintain fibroblast adherence to the surface [24, 25]. Furthermore, adhesion molecule Integrin *β*1 is a transmembrane molecule; thus, one of its functions is to act as a “binder” of extracellular matrix molecules, such as fibronectin, collagen, and vitronectin, which complement the adhesion of a cell to a surface [26]. The loss of Vinculin inhibits cell adhesion and diffusion. The absence of this protein results in lower cell dissemination on the material to be assessed and scarce formation of focal adhesions [27]; therefore, the images obtained in this study demonstrated that the group treated with MTA exhibited the lowest expression of Vinculin.

While, in this study, adherent cells, the amount of extensions, and the number of focal contacts were greater in the group of fibroblasts treated with BD than in cells treated with MTA, and this can suggest that sealants evaluated* in vitro* are biocompatible, the adhesion of cells to the surfaces of different materials reflects the relative* in vitro* biocompatibility of cement placement. Thus, one must be critical in choosing the latter, so as to ensure optimal biocompatibility.

The clinical significance of a material that is biocompatible, such as the sealants evaluated in this work (MTA and BD), is that when a suitable material is placed in contact with the extracellular space, it is activated within a short time in an accession process generated for transmembrane proteins, which ultimately affects the repair of an injury [27, 28].

Although this work is an* in vitro *study, these trials comprise an excellent tool to attempt to understand the effect of endodontic materials that appear on the market, with regard to the possible cytotoxic effect that may occur on their use.

## 5. Conclusions

Both root-end filling materials, BD and MTA, are not cytotoxic when evaluated in cultured fibroblasts of periodontal ligament in incubation periods of up to 5 days. The most biocompatible material was BD. There was an increase of cell adhesion through the expression of focal contacts observed in the case of BD, followed by MTA, but not significantly.

## Figures and Tables

**Table 1:** Relative fluorescence at 24 h.

Group	Mean ± SD	Range
Control	29.6 ± 3.7	25.5–32.7
BD	22.5 ± 1.3	21.3–24.0
MTA	11.0 ± 3.3	7.3–13.4

*p* < 0.05, BD versus MTA; data are expressed as relative fluorescence units.

**Table 2:** Expression inhibition percent of Integrin *β*1 and Vinculin.

Group	Integrin *β*1		Vinculin	
	12 h	24 h	12 h	24 h
BD	67.1	65.8	70.2	66.5
MTA	81.0	84.2	80.5	73.9
